# Middle East Respiratory Syndrome Coronavirus Transmission in Extended Family, Saudi Arabia, 2014

**DOI:** 10.3201/eid2208.152015

**Published:** 2016-08

**Authors:** M. Allison Arwady, Basem Alraddadi, Colin Basler, Esam I. Azhar, Eltayb Abuelzein, Abdulfattah I. Sindy, Bakr M. Bin Sadiq, Abdulhakeem O. Althaqafi, Omaima Shabouni, Ayman Banjar, Lia M. Haynes, Susan I. Gerber, Daniel R. Feikin, Tariq A. Madani

**Affiliations:** Centers for Disease Control and Prevention, Atlanta, Georgia, USA (M.A. Arwady, C. Basler, L.M. Haynes, S.I. Gerber, D.R. Feikin);; King Faisal Specialist Hospital and Research Center, Jeddah (B. Alraddadi);; Ministry of Health, Jeddah, Saudi Arabia (B. Alraddadi, E.I. Azhar, E. Abuelzein, A.I. Sindy, B.M. Bin Sadiq, A.O. Althaqafi, O. Shabouni, A. Banjar, T.A. Madani);; King Abdulaziz University, Jeddah (E.I. Azhar, T.A. Madani);; Ministry of National Guard, Jeddah (A.O. Althaqafi)

**Keywords:** Middle East respiratory syndrome coronavirus, disease transmission, infectious, serologic tests, RT-PCR, viruses, Saudi Arabia

## Abstract

Risk factors for human-to-human transmission of Middle East respiratory syndrome coronavirus (MERS-CoV) are largely unknown. After MERS-CoV infections occurred in an extended family in Saudi Arabia in 2014, relatives were tested by using real-time reverse transcription PCR (rRT-PCR) and serologic methods. Among 79 relatives, 19 (24%) were MERS-CoV positive; 11 were hospitalized, and 2 died. Eleven (58%) tested positive by rRT-PCR; 8 (42%) tested negative by rRT-PCR but positive by serology. Compared with MERS-CoV–negative adult relatives, MERS-CoV–positive adult relatives were older and more likely to be male and to have chronic medical conditions. Risk factors for household transmission included sleeping in an index patient’s room and touching respiratory secretions from an index patient. Casual contact and simple proximity were not associated with transmission. Serology was more sensitive than standard rRT-PCR for identifying infected relatives, highlighting the value of including serology in future investigations.

## Abstract 2

Casual contact was not associated with transmission, and serologic methods were more sensitive than real-time reverse transcription-PCR.

Middle East respiratory syndrome coronavirus (MERS-CoV) was first reported in September 2012 in a patient in Saudi Arabia (*1**,**2*). MERS-CoV is known to cause a severe acute febrile respiratory illness in humans after an incubation period of 2–14 days (*3*). As of May 1, 2016, a total of 1,728 laboratory-confirmed cases, including 624 deaths, had been reported globally (*4*); all patients have been linked to the Arabian Peninsula (*5**,**6*). Studies suggest dromedary camels as a possible natural host (*7*), although most patients report no exposure to camels (*8*). Sustained human-to-human transmission in community settings has not been observed (*6*), but transmission has been documented in healthcare settings (*9**,**10*) and in households (*11**–**14*). Specific risk factors for secondary transmission remain unknown.

In Saudi Arabia, real-time reverse transcription PCR (rRT-PCR) of nasopharyngeal or oropharyngeal swabs is used for routine MERS-CoV diagnosis and contact tracing. rRT-PCR identifies and amplifies viral RNA, indicating active infection. More recently developed serologic assays identify antibodies to MERS-CoV, indicating previous infection. MERS-CoV antibodies are rare in the general population; a nationwide serosurvey in Saudi Arabia in 2013 found antibodies in 15 (0.15%) of 10,009 persons (*15*).

MERS-CoV cases in Saudi Arabia increased substantially during March–April 2014 (*16*) in association with transmission in healthcare settings (*9**,**10*). In May 2014, as the number of urban cases decreased (*10**,**17*), a new cluster was identified 400 km south of Jeddah, in an area that had not previously reported cases. All identified patients were members of 1 extended family from the town of Al-Qouz, near Al-Qunfudah. The first MERS-CoV diagnosis was reported on May 20, 2014, in a hospitalized patient after 14 days of worsening respiratory symptoms and impending respiratory failure; by May 29, this man’s wife, brother, and nephew and the nephew’s paternal uncle had been hospitalized with confirmed MERS-CoV. These 5 relatives lived in 4 different households within Al-Qouz.

On June 4–5, 2014, representatives from the Saudi Arabia Ministry of Health (Jeddah), US Centers for Disease Control and Prevention (CDC; Atlanta, GA, USA), and King Abdulaziz University (Jeddah) joined the Al-Qunfudah Regional Health Department to investigate the family cluster. The objectives were to characterize the cluster by identifying additional cases through both rRT-PCR for viral RNA and serologic testing for MERS-CoV antibodies; to determine transmission risk factors for MERS-CoV within the affected households; and to assess possible MERS-CoV infections in the larger community, sampling both local healthcare settings and local animal workers.

## Methods

### Cluster Investigation

To find cases, we interviewed clinicians, reviewed regional records, and searched a national laboratory database. We interviewed all persons who had received a MERS-CoV diagnosis in the region and reviewed hospitalized patients’ medical charts; proxy interviews were conducted for patients who were in the intensive care unit or who had died. We then conducted a retrospective cohort study to assess infection risk factors among household members. We aimed to interview and test all members of the 4 households of the 5 known MERS-CoV–infected patients, as well as relatives who regularly visited these households and were present on the day of the on-site investigation.

On June 5, trained nurses collected 1 oropharyngeal and 1 nasopharyngeal swab for rRT-PCR and 1 blood sample for serologic testing from all available household members and visiting relatives. Hospitalized persons, persons who previously had tested positive by rRT-PCR, and children <14 years of age did not undergo serologic testing. Local public health officials had previously collected oropharyngeal swabs for rRT-PCR in the households during May 20–29; we reviewed these records. On June 5, trained physicians administered a standardized questionnaire to household members and visiting relatives to identify symptoms and healthcare exposures and infection risk factors, including animal contact, recent travel, underlying medical conditions, tobacco use, and details of exposure to each household’s index patient. An index patient was defined as the person with rRT-PCR confirmation of MERS-CoV who had the earliest date of symptom onset in the household.

### Healthcare Worker and Community Transmission

To understand whether this outbreak was affecting the broader community, we collected data at the town’s hospital, at the outpatient clinic nearest the family’s homes, at 2 local slaughterhouse facilities, and at the town’s weekly livestock animal market. All hospital staff members who had treated the first identified MERS-CoV patient from his admission on May 9 until his MERS-CoV diagnosis on May 20 underwent hospital-based rRT-PCR of oropharyngeal swabs May 21–23; serologic testing was not performed. At the outpatient clinic, all staff and a convenience sample of patients who visited the clinic on June 4 with respiratory symptoms or fever were interviewed with a standardized questionnaire and tested for MERS-CoV by using nasopharyngeal and oropharyngeal swabs for rRT-PCR and blood for serologic testing. All animal workers at 2 local slaughterhouse facilities and a convenience sample of persons with daily animal contact who were present at the town’s weekly livestock animal market on June 4 were interviewed and tested by using the same methods.

### Laboratory Testing

Specimens from hospitalized patients and hospital staff members underwent rRT-PCR at the Ministry of Health’s Jeddah regional laboratory, according to Ministry of Health protocol (*18*). Nasopharyngeal and oropharyngeal flocked swabs collected in the households, at the community clinic, and in animal workers were placed in viral transport media and transferred at 4°C to King Abdulaziz University, where rRT-PCR amplification of consensus viral RNA targets (upstream of E and open reading frame 1a) was undertaken (*19*). Serum samples were sent to CDC and screened for MERS-CoV antibodies by the recombinant MERS-CoV nucleocapsid protein ELISA, and confirmatory testing was conducted with immunofluorescence assay and microneutralization (*20*).

### Data Analysis and Ethics Review

We analyzed questionnaire data using Epi Info 7.0 (CDC, Atlanta, GA, USA). Proportions were compared by using the χ^2^ or Fischer exact test and medians by using Wilcoxon rank-sum. Risk ratios (RRs) were calculated. We compared questionnaire data for all MERS-CoV–positive (by rRT-PCR or serology) relatives >14 years of age with questionnaire data for all MERS-CoV–negative relatives >14 years of age. We excluded children from analysis because they had not had antibody testing of serum. A household secondary transmission analysis comprised relatives >14 years of age residing only in the 4 affected households. Results for MERS-CoV–positive household members who had illness onset (or tested MERS-CoV–positive) at least 2 days after the household’s index patient’s illness onset were compared with results for MERS-CoV–negative household members.

Because this investigation was part of a public health response, it was not considered by CDC and the Saudi Arabia Ministry of Health to be research that was subject to review by an institutional review board. Participants gave verbal consent.

## Results

**Figure 1:**
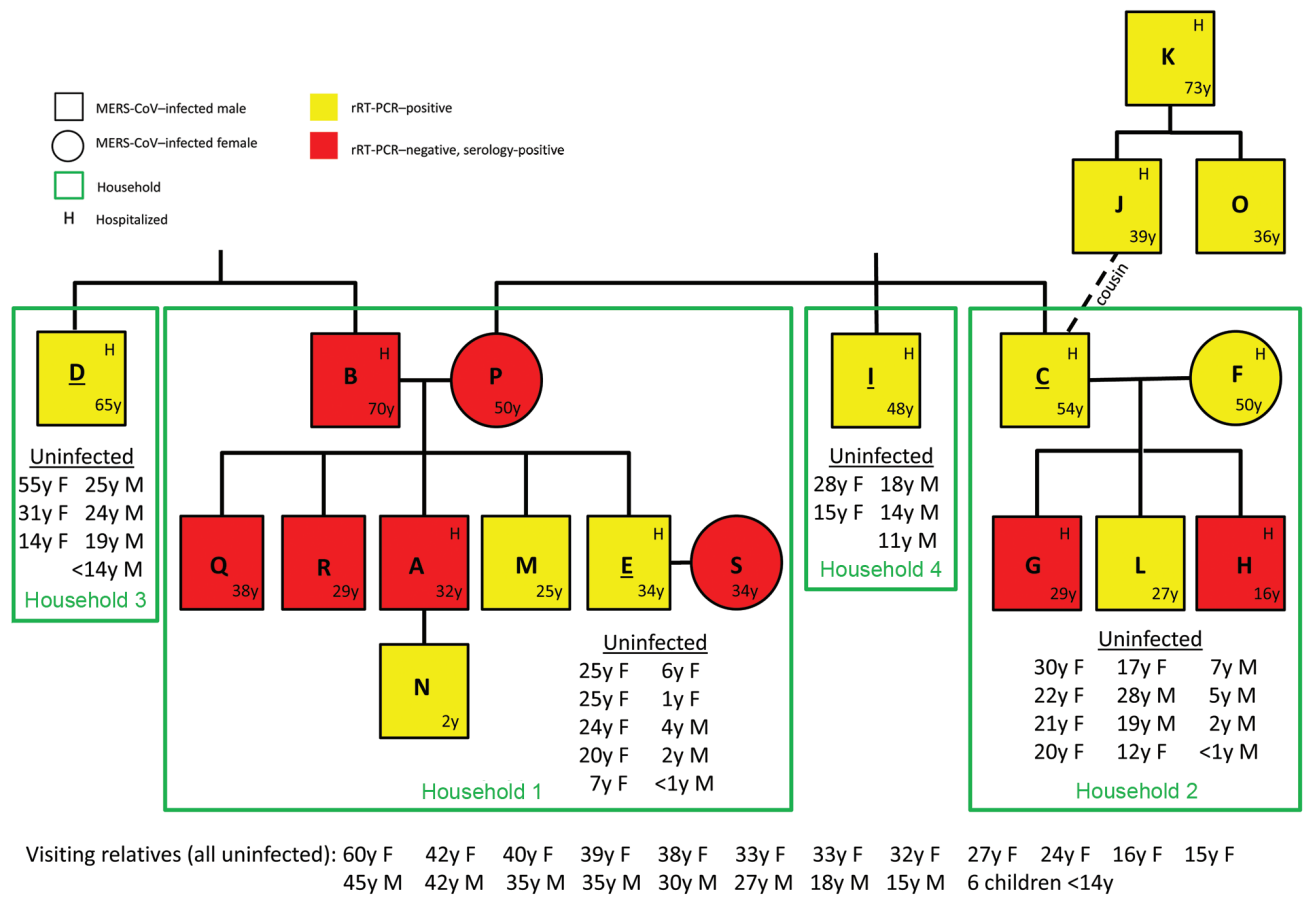
Family relationships and household distribution of persons infected with MERS-CoV, Al-Qouz, Saudi Arabia, 2014. Black lines denote standard family tree relationships. Patients are lettered in order of symptom onset or, if asymptomatic, by test date. Green boxes indicate households; all persons living in households 1–4 were tested, except for 2 adults living in household 4 (not shown). Index patient (person with earliest symptom onset diagnosed by rRT-PCR) in each household is underlined. Uninfected indicates person in household with negative rRT-PCR results and (if >14 years of age) negative serologic testing for MERS-CoV. Visiting relatives indicates extended family members who regularly visited the 4 households and were present in the households on the day of the field investigation. MERS-CoV, Middle East respiratory syndrome coronavirus; rRT-PCR, real-time reverse transcription PCR.

Nineteen extended family members had evidence of MERS-CoV by rRT-PCR or presence of MERS-CoV antibodies (Figure 1). Seventy-nine relatives were interviewed and tested for MERS-CoV by both rRT-PCR and (unless already positive by rRT-PCR or <14 years of age) serology. These persons comprised 50 (96%) of the 52 relatives living in the 4 original households (including 13 children <14 years of age); 26 relatives visiting those households (including 6 children <14 years of age); and 3 ill adults identified in a separate branch of the family tree (J, K, and O; Figure 1) after the household investigation. All 26 visiting relatives were MERS-CoV–negative by both rRT-PCR and (for adults) serology.

### Standard Diagnosis and Disease Presentation

**Figure 2:**
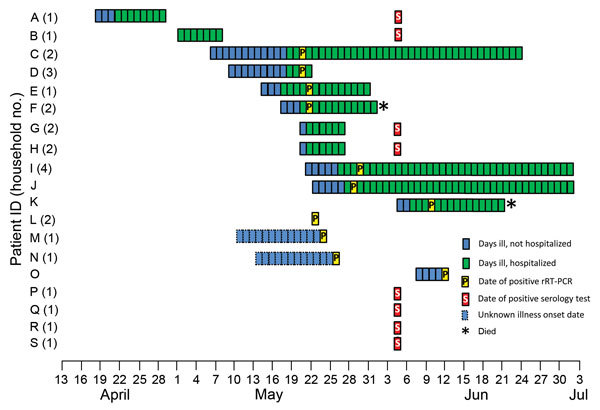
Timeline of illness onset and testing for MERS-CoV–positive family members, Al-Qouz, Saudi Arabia, 2014. Patients M and N had mild symptoms during 2 weeks before their rRT-PCR–positive results but did not identify a specific onset date; their illness dates are estimated. Patients R and S reported symptoms during the month preceding their positive serology tests but also without a specific onset date; their illness dates are not displayed. Patients L, P, and Q denied symptoms at any time. HH, household; MERS-CoV, Middle East respiratory syndrome coronavirus; Pt, patient; rRT-PCR, real-time reverse transcription PCR; S, positive serology date for rRT-PCR–negative persons.

MERS-CoV was diagnosed in 11 (58%) of the 19 patients by rRT-PCR, the standard method in Saudi Arabia (Table 1). For 7 of these, including the 5 original patients, illness was diagnosed during May 20–June 9 while they were hospitalized (Figure 2). For the other 4 patients (L, M, N, and O), MERS-CoV infection was diagnosed during May 22–June 11 through routine contact tracing and rRT-PCR by regional health officers. One of these contacts denied symptoms, 2 reported mild symptoms (i.e., cough, subjective fever) but had not sought medical care, and 1 (N, the only child given a MERS-CoV diagnosis) had visited an emergency department with fever. In the 4 households, all nonhospitalized family members were rRT-PCR–negative when tested on June 5, indicating little risk for ongoing household transmission.

**Table 1:** Clinical and laboratory characteristics of 19 family members who received a MERS-CoV diagnosis, Al-Qouz, Saudi Arabia, 2014*

Patient	Age, y/sex	Underlying condition	Symptoms			Hospitalization		Disposition	Test result	
			Presenting	Onset date		Admission date	Intubated		rRT-PCR†	Antibody‡
A	32/M	None	Fever, cough, diarrhea	Apr 18		Apr 21	No	Discharged Apr 28	Neg: Apr 21, Apr 23, Apr 24, Jun 5	ELISA >6,400; IFA pas; MNT 80
B	70/M	HTN	Fever, cough, diarrhea, shortness of breath	May 1		May 1	No	Discharged May 7	Neg: May 2, Jun 5	ELISA>6,400; IFA pos; MNT 320
C	54/M	DM, HTN, CAD	Fever, shortness of breath	May 6		May 9	Yes	Discharged Jun 23	Pos: May 20	Not tested
D	65/M	None	Fever, abdominal distention, night sweats	May 9		May 18	No	Discharged May 21	Pos: May 20 Neg: Jun 5	ELISA >6,400; IFA pos; MNT 320
E	34/M	None	Fever, cough, shortness of breath	May 14		May 17	No	Discharged May 30	Pos: May 21 Neg: Jun 5	ELISA >6,400; IFA pos; MNT 160
F	50/F	HTN, CAD	Diarrhea, fever, cough, headache	May 17		May 21	Yes	Died May 31	Pos: May 22	Not tested
G	29/M	DM	Abdominal pain, diarrhea	May 20		May 21	No	Discharged May 26	Neg: May 22, Jun 5	ELISA >6,400; IFA pos; MNT 160
H	16/M	None	Fever, cough, sore throat, diarrhea	May 20		May 21	No	Discharged May 26	Neg: May 22, Jun 5	ELISA 6,400; IFA pos; MNT 40
I	48/M	DM, HTN	Fever, cough, shortness of breath	May 21		May 26	Yes	Discharged Jul 1	Neg; May 27 Pos: May 29	Not tested
J	39/M	DM, HTN	Fever, cough, sore throat, chest pain	May 22		May 27	Yes	Discharged Jul 1	Pos: May 28	Not tested
K	73/M	DM, HTN, CAD	Fever, cough, hypoglycemia	Jun 4		Jun 6	Yes	Died Jun 20	Pos: Jun 9	Not tested
L	27/M	None	Tested as a contact; no symptoms	None		No medical care	No	Well	Pos: May 22 Neg: Jun 5	Not tested
M	25/M	None	Tested as a contact; cough	Unknown		No medical care	No	Well	Pos: May 23 Neg: Jun 5	ELISA >6,400; IFA pos; MNT 20
N	2/M	None	Tested as a contact; fever	Unknown		ED care, not admitted	No	Well	Pos: May 25 Neg: Jun 5	Not tested
O	36/M	None	Tested as a contact; subjective fever	Jun 6		No medical care	No	Well	Pos: Jun 11	Not tested
P	50/F	None	Tested as a contact; no symptoms	None		No medical care	No	Well	Neg: once during May 20–29, Jun 5	ELISA >6,400; IFA pos; MNT <20
Q	38/M	Asthma	Tested as a contact; no symptoms	None		No medical care	No	Well	Neg: once during May 20–29; Jun 5	ELISA 1,600; IFA pos; MNT 40
R	29/M	None	Fever, cough, diarrhea	Unknown		ED care, not admitted	No	Well	Neg: once during May 20–29, Jun 5	ELISA 400; IFA indeterminate; MNT 20
S	34/F	None	Fever, cough, sore throat, diarrhea, shortness of breath	Unknown		ED care, not admitted	No	Well	Neg: once during May 20–29, Jun 5	ELISA >6,400; IFA pos; MNT 20

*CAD, coronary artery disease; DM, diabetes mellitus; ED, emergency department; HTN, hypertension; IFA, immunofluorescence assay; MERS-CoV, Middle East respiratory syndrome coronavirus; MNT, microneutralization testing; neg, negative; pos, positive; rRT-PCR, real-time reverse transcription PCR. †Initial rRT-PCR for all persons was conducted at the time of illness (or among asymptomatic persons as part of routine contact tracing). ‡All serologic specimens were collected on June 5, 2014.

### Serologic Diagnosis and Disease Presentation

For 8 (42%) of the 19 positive family members, MERS-CoV infection was diagnosed only retrospectively by using serology. All 8 previously had tested negative by rRT-PCR during April 21–May 29 while hospitalized or during routine contact tracing, and all again tested negative on June 5. Two of these rRT-PCR–negative patients (A and B) had extended hospitalizations; 2 patients (G and H) had brief hospitalizations; 2 patients (R and S) had sought medical care but not required hospitalization; and 2 (P and Q) denied symptoms. Some of these patients had multiple negative tests; during an April 2014 hospitalization in Jeddah, patient A, the first patient in this family to become ill, had 3 negative rRT-PCR results of nasopharyngeal swabs.

Among the 19 relatives in whom MERS-CoV infection was diagnosed, 11 (58%) were hospitalized; 3 (16%) were treated in an emergency department for symptoms but not hospitalized; 2 (11%) reported mild symptoms but had not sought medical care; and 3 (16%) were asymptomatic. Five (26%) were intubated, 2 of whom (11%) died while hospitalized. Fever was the most commonly reported symptom (74%), followed by cough (63%), shortness of breath (44%), and diarrhea (44%). The 11 hospitalized patients were ill at home for a median of 3 days before hospital admission (range 0–9 days) (Figure 2).

### Infection Risk Factors among Adults

Fifteen (83%) of 18 MERS-CoV–positive adults were male, compared with 15 (37%) of 41 MERS-CoV–negative adults (p = 0.0009; Table 2). MERS-CoV–positive adults were more likely to have smoked sheesha, the traditional water pipe for flavored tobacco, than were MERS-CoV–negative adults (2/18 [11%] vs. 0/41; p = 0.003) and were more likely to have traveled to Jeddah (10 [56%] vs. 9 [22%]; p = 0.011) and visited a hospital there (7 [39%] vs. 5 [12%]; p = 0.019) during the month before becoming ill. MERS-CoV–positive adults were older (median age 37 years vs. 25 years; p = 0.0011) and more likely to report chronic medical problems (8 [44%] vs. 5 [12%]; p = 0.006), including diabetes mellitus and heart disease. All MERS-CoV–positive relatives denied animal contact during the 14 days before testing.

**Table 2:** Demographic, risk factor and symptom characteristics of adults with MERS-CoV–positive and MERS-CoV–negative test results in an extended family, Al-Qouz, Saudi Arabia, 2014*

Characteristic†	Test results, no. (%)		Risk ratio (95% CI)
	Positive, n = 18	Negative, n = 41	
Male sex	15 (83)	15 (37)	**4.8 (1.6–15.0)**
Reported chronic medical problem	8 (44)	5 (12)	**2.8 (1.4–5.7)**
Diabetes mellitus	5 (28)	1 (2)	**3.4 (1.9–6.1)**
Hypertension	4 (22)	3 (7)	**2.1 (1.0–4.6)**
Asthma	1 (6)	1 (2)	1.7 (0.4**–**7.1)
Heart disease	4 (22)	0	**3.1 (1.6–5.8)**
Smoked cigarettes	2 (11)	1 (2)	2.3 (0.9**–**5.7)
Smoked sheesha	2 (11)	0	**3.6 (2.4–5.4)**
Reported activities			
Visited animal market during preceding 14 d	0	2 (5)	0 (undefined)
Touched live animal during preceding 14 d	0	1 (2)	0 (undefined)
Touched camel during preceding 14 d	0	0	0 (undefined)
Traveled to Jeddah during preceding month	10 (56)	9 (22)	**2.6 (1.2–5.6)**
Visited Jeddah hospital during preceding month	7 (39)	5 (12)	**2.5 (1.2–5.0)**

Illness at any time during preceding month			
Sought medical care	13 (72)	4 (10)	**6.4 (2.7–15.2)**
Admitted to hospital	11 (61)	0 (0)	**6.9 (3.5–13.6)**
Fever	13 (72)	3 (7)	**7.0 (3.0–16.5)**
Cough	12 (67)	5 (23)	**4.9 (2.2–11.0)**
Shortness of breath	8 (44)	1 (2)	**4.4 (2.4–8.1)**
Diarrhea	8 (47)‡	3 (8)§	**3.7 (1.9–7.4)**
Vomiting	2 (12)‡	1 (3)§	**2.4 (1.0–6.0)**
Chills	5 (29)‡	1 (3)§	**3.5 (1.9–6.5)**
Body aches	9 (53)‡	1 (3)§	**5.3 (2.7–10.3)**

*Bold indicates statistical significance. Analysis includes all relatives 14 y of age tested for MERS-CoV (n = 59), regardless of household or visitor status. Positive indicates positive rRT-PCR or serologic antibody testing for MERS-CoV; negative indicates negative rRT-PCR and serologic antibody testing. Children (one 2-year-old rRT-PCR–positive child and 19 rRT-PCR–negative children) were excluded because they did not have serologic antibody testing. Listed chronic medical problems were self-reported; no one reported chronic lung or kidney disease, and other self-reported problems (hyperthyroidism, allergies, and solitary kidney) were excluded. MERS-CoV, Middle East respiratory syndrome coronavirus; rRT-PCR, real-time reverse transcription PCR.  †Ages were as follows: MERS-CoV positive: median 37 y (range 16–73 y); MERS-CoV-negative: median 25 y (range 14–60y). ‡Of the 17 persons for whom this information was reported. §Of the 40 persons for whom this information was reported.

### Household Transmission

**Figure 3:**
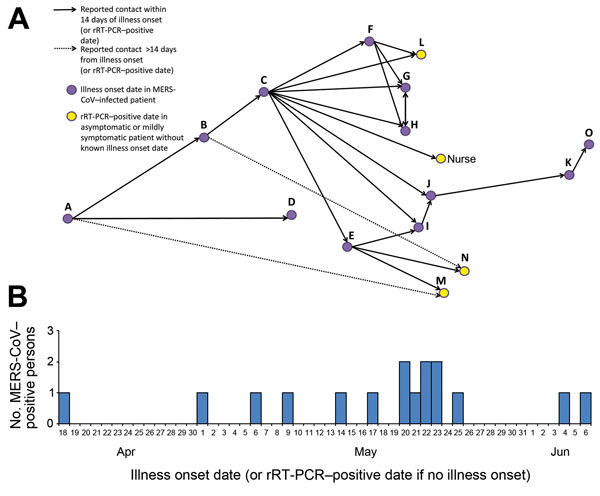
Reported contact among family members who received a MERS-CoV diagnosis and illness onset timeline, Al-Qouz, Saudi Arabia, 2014. Patients L, M, and N, as well as the infected nurse, reported no or mild symptoms and could not identify onset dates; for these 4 persons, the rRT-PCR–positive date is listed. All persons were questioned about ill family members with whom they had close contact during illness. Solid arrows indicate contact between persons within 14 days (MERS–CoV incubation period is <14 days) and indicate a likely infection source. Dashed arrows indicate contact after the 14-day incubation period; they are included for patients M and N because these patients could not identify their precise illness onset dates. MERS-CoV, Middle East respiratory syndrome coronavirus; rRT-PCR, reverse transcription PCR.

In household 1, eight of the 12 adults (a husband and wife, 5 of their adult sons, and 1 son’s wife) and 1 of the 7 children received a MERS-CoV diagnosis (household attack rate 44%; household adult attack rate 64%) (Figure 1). In household 2, five of the 12 adults (a husband and wife and 3 of their adult sons) received a MERS-CoV diagnosis (household attack rate 29%; household adult attack rate 42%). In households 3 and 4, only the index patients (both adult men) tested positive; no secondary patients were identified. All family members in whom MERS-CoV symptoms developed or who had positive rRT-PCR results reported contact with at least 1 ill relative in the preceding 14 days (Figure 3). 

When we compared results for the 9 secondary adult patients (adults who tested MERS-CoV–positive with illness onset after the presumed index patient) in these 4 households with the results for 21 adults in the households who tested negative, we identified several major risk factors for MERS-CoV transmission in univariate analysis (Table 3). These risk factors included sleeping in the same room as an index patient (RR 4.1, 95% CI 1.5–11.2), touching his respiratory secretions (RR 4.0, 95% CI 1.6–9.8), and removing his biological waste (RR 3.2, 95% CI 1.2–8.4). Notable variables not associated with being a secondary patient included hugging or social kissing; sharing plates, cups, meals, sheesha, or a toilet; and cleaning or feeding the index patient.

**Table 3:** Exposures to MERS-CoV index patients by household adult members with and without secondary MERS-CoV infection living in 4 households, Al-Qouz, Saudi Arabia, 2014*

Exposure/activity	Infected by secondary transmission, no. (%), n = 9	Uninfected, no. (%), n = 21	Risk ratio (95% CI)
Daily household activities			
Treated index patient during time he was ill at home before hospitalization	8 (89)	13 (62)	3.4 (0.5–23.5)
Shared meal	6 (67)	11 (52)	1.5 (0.5–5.0)
Ate from same plate with hands	6 (67)	8 (38)	2.3 (0.7–7.5)
Hugged	7 (78)	8 (38)	3.5 (0.9–14.2)
Kissed	7 (78)	9 (43)	3.1 (0.8–12.4)
Shook hands	6 (67)	11 (52)	1.5 (0.5–5.0)
Shared drinking cup	4 (44)	9 (43)	1.0 (0.3–3.1)
Shared sheesha	0	0	Undefined
Shared utensils	1 (11)	7 (33)	0.3 (0.1–2.3)
Slept in same room	5 (56)	2 (10)	**4.1 (1.5**–**11.2)**
Shared toilet	4 (44)	6 (29)	1.6 (0.5–4.7)

Caregiving activities			
Helped care for index patient at home	6 (67)	8 (38)	2.3 (0.7–7.5)
Changed or washed clothes, sheets	5 (56)	4 (19)	**2.9 (1.0–8.4)**
Cleaned index patient	4 (44)	5 (15)	2.6 (0.9–7.3)
Cleaned in room	4 (44)	4 (19)	2.2 (0.8–6.2)
Administered medicine	5 (56)	6 (29)	2.2 (0.7–6.4)
Fed index patient	5 (56)	7 (33)	1.9 (0.6–5.6)
Touched index patient’s respiratory secretions	4 (44)	1 (5)	**4.0 (1.6**–**9.8)**
Removed index patient’s waste	4 (44)	2 (10)	**3.2 (1.2**–**8.4)**

Proximity to index patient			
Within 1 m during time he was sick at home	8 (89)	12 (57)	4.0 (0.6–27.8)
Within 1 m every day	7 (78)	9 (43)	3.1 (0.8–12.4)
Within 1 m on day preceding hospitalization	7 (78)	11 (52)	2.3 (0.6–9.3)
Visited index patient in the hospital	6 (67)	10 (48)	1.8 (0.5–5.7)

*Bold indicates statistical significance. This household transmission analysis included relatives >14 y of age living in the 4 households of the index patients, defined as the first patient in the household who received a MERS-CoV diagnosis by rRT-PCR. Secondary transmission is defined as onset of illness or testing positive for MERS-CoV after the household’s index patient had received a diagnosis. Two MERS-CoV–infected household members were excluded from analysis because they had illness onsets before the presumed household index patient’s illness and were subsequently reported to have MERS-CoV antibodies. MERS-CoV, Middle East respiratory syndrome coronavirus; rRT-PCR, real-time reverse transcription PCR.

### Community Transmission

Except for members of this extended family, the regional hospital admitted no other MERS-CoV patients. Of 131 hospital workers who cared for patient C, 1 (0.8%), a nurse who remained asymptomatic, tested positive by rRT-PCR on May 23. All 44 persons tested at the outpatient clinic (21 patients with respiratory complaints and 23 staff) were MERS-CoV–negative by both rRT-PCR and serology. All 11 slaughterhouse workers and 10 livestock market participants tested negative by rRT-PCR. One (5%) asymptomatic slaughterhouse worker demonstrated antibodies to MERS-CoV by serology. He had no known contact with any family members in the cluster.

## Discussion

This investigation defined the epidemiology of a large family cluster of MERS-CoV infection in Saudi Arabia, identified multiple possible household transmission risk factors, and highlighted the useful role of serology in describing the extent of family clusters and spectrum of illness. For approximately half (42%) of the 19 MERS-CoV–infected family members, rRT-PCR results were negative while they were ill or after recognized exposure, and infection was diagnosed only retrospectively by serology; this included patients tested during extended hospitalizations and demonstrates real-world limitations in rRT-PCR or timing of specimen collection, transport, and testing. This finding highlights the need for clinicians to consider MERS-CoV diagnoses in appropriate clinical settings, even in patients with negative rRT-PCR results. Clinicians should consider obtaining lower respiratory tract specimens to improve the sensitivity of rRT-PCR, particularly if nasopharyngeal and oropharyngeal test results are negative and clinical suspicion is high, and they should consider follow-up serologic testing. Most importantly, clinicians should apply appropriate infection control practices for patients with clinically suspected illness, regardless of initial rRT-PCR results.

Only 3 of the 19 MERS-CoV–infected family members were women, all wives of patients. Infection predominance in males has characterized MERS-CoV since its identification (64% of patients globally have been male [*5*]) and might reflect biologic or behavior differences, such as men and women socializing separately (*21**,**22*). Underlying illness has previously been linked to more severe MERS-CoV symptoms and signs (*23*), but whether underlying illness also makes persons more susceptible to initial MERS-CoV infection is less clear. This study, in which 96% of household members were tested, found an increased infection risk among persons with underlying chronic illnesses.

Our data indicate close contact (e.g., sleeping in the same room as an index patient) and direct patient care activities (e.g., touching a patient’s respiratory secretions and removing his body waste), rather than casual contact or simple proximity, increases risk for transmission. Although smoking sheesha was a statistically significant risk factor for infection, the 2 infected family members who smoked sheesha denied smoking together, making it an unlikely mechanism of transmission. Guidance on preventing household transmission of MERS-CoV should emphasize minimizing close contact with patients. Outside of this extended family (and 1 asymptomatic exposed nurse and 1 asymptomatic camel slaughterhouse worker), we did not find evidence for wider community transmission of MERS-CoV.

Two (11%) of the infected family members died. As of May 1, 2016, Saudi Arabia had reported 588 deaths among 1,380 confirmed MERS-CoV patients, for an overall 43% case-fatality rate (*17*). The substantially lower fatality rate in this family most likely reflects aggressive contact tracing and use of serology to identify mildly symptomatic and asymptomatic patients. Patients in this family also were younger (median age 37 years) than MERS-CoV patients globally (median 48 years [*5*]). The case-fatality rate in this cluster might reflect the broader population across the spectrum of illness.

Previously described MERS-CoV family clusters and household contact investigations have reported household attack rates ranging from <1% to 19% (*11**–**14*). Household attack rates in this investigation were markedly higher; 64% and 42% of the adults in 2 households were infected. This difference could be due to methodologic differences in our investigation; serology identified mildly symptomatic and asymptomatic patients, which would increase the attack rate over investigations that relied only on rRT-PCR. The attack rate, however, could have actually been higher in this cluster for several reasons. First, MERS-CoV diagnoses were missed or delayed among the first cases in the family. The first 2 patients to become ill (patients A and B) were hospitalized but had negative rRT-PCR results during illness; their subsequent positive serologic results confirmed that the earlier illness had in fact been undiagnosed MERS-CoV. The third patient (patient C) was ill for 14 days before receiving a diagnosis, a time during which many other family members reported contact with him. Because this community had not previously experienced MERS-CoV infections, family members and local hospital staff might have had limited suspicion for MERS-CoV infection and not limited close contact. In contrast, when MERS-CoV infection was diagnosed in index patients in households 3 and 4, the family (and local hospital staff) was highly attuned to the possibility of MERS-CoV infection and took precautions to prevent its spread.

Second, patient C might have been part of a super-spreading event because up to 8 other infected persons might have been infected through contact with him (Figure 3). The concept of a super-spreading event was described during the outbreak of severe acute respiratory syndrome coronavirus in 2003 (*24**,**25*) and more recently was observed in the MERS-CoV outbreak in healthcare facilities in South Korea, where each of 3 patients was associated with infection in >20 other persons (*26**,**27*). Finally, although all infected persons denied animal contact and the range of symptom onset dates indicated ongoing person-to-person spread, an environmental point-source of infection might have been missed.

Our study had several limitations. First, in household 1, two persons had, in fact, been ill with MERS-CoV before the presumed household index patient received a diagnosis. These persons had negative rRT-PCR results while ill, and infection was diagnosed retrospectively when later serologic test results were positive. The study questionnaire focused primarily on household exposures to the presumed index patient, but persons in this household might have had a range of exposures to all 3 persons, making isolation of the specific exposure that resulted in secondary infection more difficult. Second, the small sample size did not enable multivariable risk factor analysis and confounding and collinearity could not be evaluated. Third, serologic testing was not conducted for children <14 years of age, and they were excluded from risk factor analysis; the observed lower incidence of infection among children could be investigated by including children in future serologic investigations. Fourth, sequential serologic testing was not performed, so it is possible that persons identified as MERS-CoV-negative might not yet have seroconverted, although none had developed respiratory illnesses before or after testing. Finally, specimens were not available for genome sequencing, which might have helped to clarify transmission chains within the family. 

More studies are needed to define the virologic and epidemiologic factors involved in household transmission of MERS-CoV to inform future public health response. Including serologic methods in these investigations will help better identify the spectrum of MERS-CoV clinical presentations. As testing methods evolve, maintaining strict infection control practices for ill patients with strong epidemiologic risk factors for MERS-CoV remains crucial to containing further spread.
